# Insight into the Role of Epigenetic Processes in Abiotic and Biotic Stress Response in Wheat and Barley

**DOI:** 10.3390/ijms21041480

**Published:** 2020-02-21

**Authors:** Lingyao Kong, Yanna Liu, Xiaoyu Wang, Cheng Chang

**Affiliations:** 1 College of Life Sciences, Qingdao University, Qingdao 266071, China; konglingyao@126.com (L.K.); Lyn19801259593@163.com (Y.L.); 2017020884@qdu.edu.cn (X.W.); 2 National Key Facility for Crop Gene Resources and Genetic Improvement, Institute of Crop Science, Chinese Academy of Agricultural Sciences, Beijing 100081, China

**Keywords:** epigenetic, abiotic and biotic stress, wheat, barley, DNA methylation, histone modification, chromatin remodeling, non-coding RNAs

## Abstract

Environmental stresses such as salinity, drought, heat, freezing, heavy metal and even pathogen infections seriously threaten the growth and yield of important cereal crops including wheat and barley. There is growing evidence indicating that plants employ sophisticated epigenetic mechanisms to fine-tune their responses to environmental stresses. Here, we provide an overview of recent developments in understanding the epigenetic processes and elements—such as DNA methylation, histone modification, chromatin remodeling, and non-coding RNAs—involved in plant responses to abiotic and biotic stresses in wheat and barley. Potentials of exploiting epigenetic variation for the improvement of wheat and barley are discussed.

## 1. Introduction

As two founding crops of the agricultural revolution that took place 10,000 years ago in the Fertile Crescent, bread wheat (*Triticum aestivum* L. ssp. *aestivum*) and cultivated barley (*Hordeum vulgare* L. ssp. *vulgare*) are widely cultivated in the world and provide more than 20% of the caloric intake for one-half of the world’s population [1,2,3,4]. There is a crucial need to improve the production of wheat and barley for a growing population. However, environmental stresses such as salinity, drought, heat, freezing, heavy metal and even pathogen infections seriously threaten the growth and yield of wheat and barley under field conditions [5,6,7,8,9,10,11,12]. For instance, the majority of crops are highly sensitive to salinity, and the average yield of all important glycophytic crops decreased by 50%–80% at medium salinity conditions [5]. The accumulation of salt in the soil solution reduces the absorption of water and nutrients, leading to osmotic stress, ion toxicity, nutrient imbalance, and even water deficit [6,7]. More than 5% of Na^+^ can cause the clay to expand excessively when wet, severely restricting the movement of air and water, then resulting in poor drainage [7]. Currently, of the 230 million hectares (ha) of irrigated land in the world, 45 million ha (19.5%) have been threatened by salinity [7]. As global climate conditions continue to deteriorate, drought and heat always go hand in hand, which leads to higher crop losses. For barley, Xie et al. reported that the yield could be reduced by between 3% and 17% in those harsh conditions [8]. For wheat, the optimum temperature is about 21 °C at reproductive growth stage. Temperatures in excess of 33 °C in this stage result in a decrease of leaf photosynthesis, an accumulation of peroxides, and serious yield loss [9]. For heavy metal, cadmium (Cd) at low concentrations (0.3–0.8 mg kg ^−1^) in soils could inhibit regular cell division, decrease photosynthesis and impair antioxidant enzyme activity [10,11]. In all major wheat-growing areas, lead (Pb) accumulation is generally accompanied by Cd pollution, seriously threatening crop yield and safety [12]. In acid soils, aluminum (Al) ions severely inhibit root growth and reduce the absorption of water and nutrients, resulting in crop yields [13]. In addition to these abiotic stresses, biotic stress also seriously damage grain yield and quality. It has been conservatively estimated that fungal pathogens alone are responsible for 15% to 20% yield losses per annum [14,15]. Among them, rust, the blotches and head blight/scab are the most devastating diseases leading to great yield loss in bread wheat [15]. Therefore, how to improve plant resistance against abiotic and biotic stresses in wheat and barley is the focus of attention for breeders.

Through evolution, plants have acquired highly sophisticated systems to cope with various environmental stresses. The past decade has seen unprecedented progress in understanding the signaling pathways controlling the plant responses to stresses, which has been summarized in prior reviews [16,17,18]. Activation of these signaling pathways usually results in the dramatic transcriptional reprogramming to initiate a set of stress responses [19,20,21]. There is increasing evidence indicating that this transcriptional reprogramming and regulation of stress-responsive genes involves diverse epigenetic processes and elements, such as DNA methylation, histone modification, chromatin remodeling and non-coding RNAs [22,23,24]. Here, we summarized the most recent progress on studies of epigenetic regulation of plant responses to abiotic and biotic stresses in wheat and barley, and discussed the potentials of exploiting natural and induced epigenetic variation for the improvement of wheat and barley.

## 2. DNA Methylation

As a type of DNA chemical modification, DNA methylation regulates the chromatin structure, DNA stability, and even gene expression without changing the DNA sequence. Under the action of DNA methyltransferase, the cytosine C5 position is covalently bonded with a methyl group, which is one of the most common modifications of DNA in eukaryotic cells [25,26,27]. In plants, cytosine methylation is detected in the context of CG, CHG, and CHH (where H is any nucleotide except G) [28,29], in which CG is the most abundant and widespread methylation site [30]. It has been revealed that the DNA de novo C methylation in *Arabidopsis* is catalyzed by methyltransferase DOMAINS REARRANGED METHYLTRANFERASE 1 (DRM1) and DRM2 [29], while the maintenance of DNA methylation in mitosis and meiosis relies on the METHYLTRANSFERASE 1 (MET1) [31], CHROMOMETHYLASE 2/3 (CMT2/3) [27,31]. High-resolution DNA methylation profiling in *Arabidopsis* and rice revealed that DNA methylation could take place in many chromatin regions, including intergenic transposable elements (TE), gene promoters and even gene-body [32,33,34,35]. Many studies revealed that DNA methylation in TEs is required for maintaining genome integrity [32,33,34,35]. Furthermore, DNA methylation at promoters generally represses gene expression, whereas methylation in gene-body DNA appears to be associated with active gene expression in *Arabidopsis* [32,33,34,35].

As an important epigenetic process, DNA methylation generally regulates plant responses to environmental stresses such as salinity and heavy metal stress in wheat and barley [36,37]. For instance, a recent report showed that DNA methylation could regulate the expression of a salinity-responsive gene in bread wheat [25]. A reduction in global DNA methylation level was observed in two bread wheat cultivars (salinity tolerant wheat cultivar SR3 and salinity susceptible wheat cultivar JN177) upon exposure to salinity stress [25]. Notably, the methylation level at the promoter of a stress-responsive gene TaFLS1 (encoding a flavonol synthase) was lower in the salinity tolerant wheat cultivar SR3 than in salinity susceptible wheat cultivar JN177, which is opposite to the higher gene expression in SR3 than in JN177, suggesting that DNA methylation might get involved in regulation of wheat salinity tolerance [25]. Besides, the modulation of metal-stress response by DNA methylation is reported in wheat and barley [37,38]. In heavy metal detoxifications, the multidrug resistance-associated protein (MRP) type ATP-binding cassette (ABC) transporters play important roles, which also involve in other plant biological processes such as pathogen response and development [39,40,41,42]. In addition, AtABCC5 also get involved in drought stress response by altering guard cell movement in *Arabidopsis* [43,44]. Eighteen MRP-type ABC transporter genes were identified from the wheat genome [41]. Shafiq et al. found that the expression of TaABCCs and HEAVY METAL ATPASE 2 (TaHMA2) was higher in the heavy metal-resistant wheat varieties than in heavy metal-sensitive varieties upon exposure to the heavy metal stresses [37]. Furthermore, DNA methylation levels at the promoter of TaABCCs and TaHMA2 were lower in heavy metal-resistant varieties than in heavy metal-sensitive varieties under heavy metal stresses, suggesting that DNA methylation is associated with metal stress tolerance in wheat [37]. In barley, Al-activated citrate transporter1 (HvAACT1) is a major gene in charge of citrate efflux from roots for external Al detoxification in the rhizosphere [45,46]. Although the expression of HvAACT1 was not altered by Al treatment, its expression was significantly higher in Al-tolerant accessions than in Al-sensitive accession, indicating that HvAACT1 is a principal gene regulated Al tolerance in barley [47]. It is intriguing that in several European barley accessions, DNA methylation level at a multiretrotransposon-like (MRL) sequence, localized at the upstream genomic sequence of HvAACT1, is associated with the expression of HvAACT1 [38]. DNA demethylation in MRL resulted in the enhanced expression of HvAACT1, especially in the zone of root apical [39]. Meanwhile, low-level expression of HvAACT1 was found associated with a higher degree of DNA methylation in MRL, suggesting that the DNA methylation regulates the HvAACT1 expression, which was responsible for Al tolerance in barley [38]. Compared with extensive studies on the role of DNA methylation in regulation of abiotic stress tolerance, understanding of DNA methylation regulating defense response to pathogens is limited. A recent study reported that DNA methylation, particularly CHH methylation, gets involved in the regulation of defense responses to *Bgt* (*Blumeria graminis* f. sp. *tritici*), the causal agent of wheat powdery mildew, in wheat diploid progenitor *Aegilops tauschii* [48]. Upon Bgt infection, abundant differentially methylated regions (DMRs) were associated with CHH hypomethylation [48]. WGBS (whole-genome bisulfite sequencing) further revealed that TAGs (genes near transposable elements) with CHH-hypomethylated DMRs were enriched in genes with annotation for ‘response to stress’ functions, such as receptor kinase, peroxidase, and pathogenesis-related genes, suggesting that DNA methylation is involved in the regulation of plant defense responses in crops [48]. In addition, several instances indicated that the sensitivity of transcription factors (TFs) to DNA methylation can affect the binding of TF to chromatin [49]. In *Arabidopsis*, O’Malley et al. found that a regulatory relationship may exist between specific DNA methyltransferase and TF family [49]. Although similar results have not been found in wheat and barley, it is important for us to understand how DNA methylation plays a vital role in plant responses to stress.

To balance the genomic methylation level and fine-tune gene expression, DNA demethylases were employed to remove 5-methylcytosine and replace it with unmethylated cytosine [50]. In plants, DNA demethylation occurs in two ways: passive demethylation and active demethylation. During DNA replication, methylated cytosines are replaced with unmodified cytosines in passive demethylation [51,52]. Active DNA demethylation is mediated by specific DNA glycosylases, which hydrolyze the N-glycosidic bond between ribose and base [51,52]. In the past decade, several DNA glycosylases, including DMEMER (DME) and REPRESSOR OF SILENCING 1 (ROS1), involved in the active DNA demethylation were well studied in the model plant *Arabidopsis* [53,54]. Recently, exploration on the DNA glycosylase in wheat and barley has also emerged. For instance, *HvDME* encodes a DME-family DNA glycosylase in barley (Table 1) [55]. The expression of *HvDME* is markedly induced in drought-treated barley seedlings, especially in the drought-tolerant cultivars, suggesting a potential role of DNA demethylation in the regulation of barley responses to drought stress (Table 1) [55].

## 3. Histone Modification

As one of the most common types of epigenetic regulation, diverse histone modification manners have been characterized, including acetylation, methylation, butyrylation, propionylation, crotonylation, malonylation, succinylation, 2-hydroxyisobutyrylation and β-hydroxybutyrylation [61,62,63]. Among these histone modifications, histone methylation/demethylation and acetylation/ deacetylation have been widely studied, which regulates many biological processes in plants, including development and responses to biotic and abiotic stresses [62,63]. The majority of histone methylation takes place on the lysine residue of histone H3, such as H3K4me3, H3K36me3, H3K79me3, H3K9me2 and H3K27me3, in which H3K4me3 and H3K27me3 are highly conserved epigenetic marks for gene activation and repression, respectively [64,65,66]. Histone methylation is dynamically regulated by the histone methyltransferases (HMTs) and histone demethylases (HDMs) [64,65,66]. For instance, the repressive H3K27me3 modification is mediated by HMT complexes PRC1 and PRC2 recruited by various DNA-binding proteins. The first HMT recruiters in *Arabidopsis* are the members of the BBR/BPC family, that were shown to be responsible for establishing the silencing mark [67,68]. As another common type of histone modification—histone acetylation—is reversible and dynamically maintained by the antagonistic action of histone acetyltransferases (HATs) and histone deacetylases (HDACs) [56,69]. It is generally realized that the acetylation neutralizes the positive charge of lysine side chains on histones and reduces its interaction with the negatively charged DNA backbone, and thus relax the chromatin structure and promote gene transcription [70,71]. Indeed, H3K4ac and H3K9ac are often associated with gene activation, thereby modulating numerous biological processes such as stress responses in higher plants such as model plant *Arabidopsis* [72,73,74]. Increasing evidence from studies in *Arabidopsis* revealed that histone acetylations such as H3K4ac and H3K9ac are usually connected with histone methylation including H3K4me3, simultaneously regulating gene expression [71]. Therefore, histone post-transcriptional modifications are cross-talked, which fine-tunes the gene expression and response in various important biological processes in eukaryotes [75].

Previous studies revealed that various HATs and HDACs modulate plant gene expression in response to environmental stresses in wheat and barley. For instance, TaGCN5, a wheat ortholog of *Arabidopsis* histone acetyltransferases AtGCN5, plays an important role in regulating wheat defense response to heat and salt stresses (Table 1) [56,57]. The expression of *TaGCN5* was induced by treatment with heat and salt in bread wheat (Table 1) [56,57]. In *Arabidopsis*, GCN5 protein is recruited to the promoters of *HSFA3* and *UVH6* (UV-HYPERSENSITIVE 6) in response to heat stress, and enriched at the promoters of *CTL1*, *PGX3* and *MYB54* (involved in tolerance of salt stress) under salt stress as well [56,57]. At the same time, GCN5 was revealed to facilitate the acetylation of H3K9 and H3K14, which is associated with activation of *HSFA3, UVH6*, *CTL1*, *PGX3*, and *MYB54* in *Arabidopsis* [56,57]. Interestingly, the expression of *HSFA3, UVH6*, *CTL1*, *PGX3*, and *MYB54* were constitutively increased in *35S:TaGCN5/gcn5* transgenic *Arabidopsis*, compared with wild-type and *gcn5* plants, suggesting that GCN5-mediated histone acetylation responding to abiotic stress tolerance might be conserved in *Arabidopsis* and bread wheat (Table 1) [56,57]. In addition to abiotic stresses, biotic stresses such as the fungal infection also initiate the plant responses partly controlled by histone modifications. Recently, Sharma et al. characterized the wheat histone acetylation at the promoters of defense-related genes upon infection of *Puccinia triticina,* the causal agent of wheat leaf rust. In this study, two near-isogenic wheat lines (NILs), leaf rust-susceptible NIL and resistant NIL, were employed and the expression levels of six defense-related genes were analyzed as well [73]. Among the six genes, *N-acetyltransferase* is activated by enrichment of H3K4ac and H3K9ac at its promoter in leaf rust-susceptible NIL, and repressed by the histone deacetylation in leaf rust-resistant NIL [71]. In contrast, enrichment of H3K4ac and H3K9ac are largely correlated with higher expression of *Peroxidase 12* in both NILs. The expression of other remaining four genes (*WRKY 70*, *ASR1*, *Peroxidase 12* and *Sarcosine oxidase*) was not correlated with histone acetylation [67,73]. These results suggested that histone acetylation indeed get involved in the regulation of wheat response to *P. triticina* infection, whose underlying mechanism remains further study. Recently, the wheat histone deacetylase TaHDA6 was found to interact with the wheat WD40-repeat protein TaHOS15 and was recruited to the promoters of defense-related genes, where TaHDA6 mediated histone deacetylation (Table 1) [58]. The decrease of the transcription level of TaHDA6 results in the enhanced transcription of defense-related genes, thus strengthening resistance to *Bgt* infection, suggesting that TaHDA6 fine-tunes the acetylation levels of these wheat defense-related genes (Table 1) [58]. In barley, the senescence-associated gene *HvS40* exhibited enhanced H3K9ac at its promoter and coding regions during the early response to drought stress [76]. Interestingly, histone modifications such as histone methylation and acetylation were found usually accompanied by DNA methylation in response to environmental stresses in the model plant *Arabidopsis* [77,78]. However, it remains to be studied about the interplay of histone modifications with other epigenetic regulation such as DNA methylation in governing resistance against environmental stresses in wheat and barley.

## 4. Chromatin Remodeling

Besides DNA methylation and histone modifications, chromatin structure and gene expression may also be affected by chromatin remodeling, a process that disrupts histone-DNA interactions resulting in the altered accessibility of specific DNA regions to transcription machinery [79,80]. Chromatin remodeling factor (CHR), including the SWI/SNF ATPases, the imitation switch (ISWI) ATPases, and the chromodomain and helicase-like domain (CHD) ATPases subfamilies, could mediate either the ATP-dependent chromatin remodeling or the posttranslational histone modifications [57,58,73,74,75,76,77,78,79,80,81,82,83,84]. The ATP-dependent chromatin remodeling complexes could alter nucleosome composition, and positioning and thus regulate DNA accessibility and gene expression. In contrast, the posttranslational histone modifications could alter the interaction between nucleosomes, and thus affect the chromatin compactness and structure in model plant *Arabidopsis* [85,86,87]. Chromatin remodeling has been well documented to regulate plant growth, development and response to environmental stresses in *Arabidopsis* and rice [88,89]. Besides, the investigation on the role of chromatin remodeling in regulating plant responses to stresses in wheat and barley is emerging [88,89]. For instance, the wheat CHD-type chromatin remodeling factor TaCHR729 was reported recently to interact with the *TaKCS6* promoter-associated bHLH type transcription factor 1 (TaKPAB1) and thereby bind to the promoter regions of wheat *3-KETOACYL-CoA SYNTHASE* (*TaKCS6*), which encodes a key enzyme in the wheat cuticular wax biosynthesis (Table 1) [59]. Interestingly, TaCHR729 was found to promote H3K4me3 at the promoter region of *TaKCS6* and positively regulate the *TaKCS6* transcription (Table 1) [59]. Consistently, silencing of *TaCHR729* attenuated the biosynthesis of wheat cuticular wax and germination of *Bgt* conidia, suggesting that the wheat chromatin remodeling factor TaCHR729 regulate the wheat-powdery mildew interaction through mediating histone methylation and fine-tuning the cuticular wax biosynthesis (Table 1) [59].

Although the study of chromosome remodeling in response to stress in wheat and barley is very limited, the research in *Arabidopsis* is abundant, in-depth and worthy reference. For instance, ABA signal transduction which responds to abiotic stresses, such as drought, salinity and freezing, is regulated by chromatin remodeling in *Arabidopsis*. The clade A PP2C phosphatase HAB1 (HYPERSENSITIVE TO ABA 1, a core component in ABA signal pathway) interacts with SWI3B physically, a core subunit of the putative SWI/SNF complex in *Arabidopsis* [90]. As a phosphatase, HAB1 may directly dephosphorylate SWI/SNF complexes including SWI3B in an ABA-dependent manner [90]. Another study has shown that several chromatin regulators, such as BRM SWI/SNF ATPase, could be phosphorylated by of SnRK2 type kinases (another core component in ABA signal pathway) as the substrates [91,92]. These results suggest that the phosphorylation and dephosphorylation states of SWI/SNF complexes may modulate the response to environmental stresses by ABA signal pathway and further molecular mechanisms need to be studied in wheat and barley.

## 5. Non-coding RNAs

As important epigenetic elements, non-coding RNAs (ncRNAs) widely regulate plant multiple processes, including growth, development and even responses to environmental stresses. Based on the size, ncRNA can be divided into short-chain non-coding RNAs and long-chain non-coding RNA [93,94,95]. In the past few decades, enormous studies in animals and plants have revealed that short-chain non-coding RNAs, such as microRNAs (miRNA) and small interfering RNAs (siRNAs), participate in both transcriptional and post-transcriptional regulation of gene expression [93,94,95]. MicroRNAs and siRNAs usually contain 18-24 nucleotides (nt) and were classified as small RNA (sRNA). In contrast, non-coding RNA with more than 200 nucleotides (nt) length has been generally defined as long non-coding RNAs (lncRNAs) [93,95,96]. In eukaryotic cells, miRNAs coding genes are transcribed to generate primary miRNAs (pri-miRNAs), which are then cleaved and processed into mature miRNAs under the action of DICER-LIKE proteins (DCLs). In *Arabidopsis*, ARGONAUTE (AGO) family proteins such as AGO1 then bind the nascent mature miRNAs and guide the target-specific post-transcriptional gene silencing (PTGS) [97]. Unlike microRNA, short/small interfering RNAs (siRNAs) are generated from long linear double-stranded RNAs with 20-24 nt length and are transcribed by RNA polymerase IV (RNAPIV) from transposons and repetitive regions. So far, several subclasses of siRNAs have been identified, some of which function in PTGS and others function in transcriptional gene silencing (TGS) [98,99]. LncRNAs are widespread in all species and take part in gene expression regulation at transcription and post-transcription, epigenetic level [100,101]. LncRNAs share similarities with mRNAs in the structure and biogenesis process, and they are transcribed by RNA polymerase II (RNAPII) and poly-adenylated [102]. Like mRNAs, lncRNAs own multiple exons and are subjected to alternative splicing. However, lncRNAs are short of discernable coding potential [102]. More recently, lncRNAs are revealed to have adjusting functions in the major biological processes, including development, vernalization, and environmental stress adaptation by direct and indirect manners in *Arabidopsis* [103,104].

Increasing evidence from *Arabidopsis* studies revealed that ncRNAs such as siRNAs and lncRNAs regulate plant stress-responsive gene expression through multiple epigenetic mechanisms, including DNA methylation, histone modification and genome topology changes [97]. For instance, siRNAs and lncRNAs both participate into the DNA de novo cytosine methylation via the RNA-directed DNA methylation (RdDM) pathway in *Arabidopsis* [105]. *Arabidopsis* RNAPIV-generated siRNAs could load to Argonaute 4 (AGO4) and interact with lncRNAs generated by RNAPII to constitute a siRNA–AGO4–lncRNA silencing complex, which subsequently recruits the DMT domains rearranged methyltransferase 2 (DRM2) to mediate DNA de novo cytosine methylation [106]. *Arabidopsis* mutant deficient in NRPD2, an essential subunit of RNAPIV were hypersensitive to heat stress, suggesting that RdDM pathway is essential to the regulation of plant stress responses [107]. Besides, some lncRNAs were revealed to regulate histone modifications in *Arabidopsis* [108]. For instance, the cold-induced lncRNA *COOLAIR*, a group of long antisense RNAs expressed from the *FLOWERING LOCUS C* (*FLC*) locus, promote the replacement of H3K36me3 with H3K27me3, as well as the H3K4me2 demethylation, at *FLC* locus during cold exposure [109,110]. Similarly, another cold-induced lncRNA *COLDAIR* could interact with polycomb repressive complex 2 (PRC2) to facilitate H3K27me3 enrichment at *FLC* [111,112,113]. In addition to DNA methylation and histone modification, genome topology is also regulated by ncRNAs in *Arabidopsis*. For instance, *Arabidopsis* lncRNA APOLO promotes the chromatin loop formation at the *PINOID* (*PID*) locus, encoding a key regulator of polar auxin transport, which is further regulated by RdDM and ultimately determines the *PID* expression patterns [114]. With the development of high-throughput sequencing technology and computational methods, the research of ncRNA has been carried out gradually in wheat and barley [60,115,116,117,118]. For instance, Zhang et al. found that four lncRNAs (*TalncRNA18*, *TalncRNA73*, *TalncRNA106*, and *TalncRNA108*) exhibited differential expression upon infection of *Puccinia striiformis* f. sp. *tritici* (*Pst*), suggesting that these lncRNAs may get involved in regulation of wheat defense responses to *Pst*. However, detailed epigenetic mechanism in the regulation of wheat and barley stress responses remain to be explored in the future research.

## 6. Concluding Remarks

**Figure 1:**
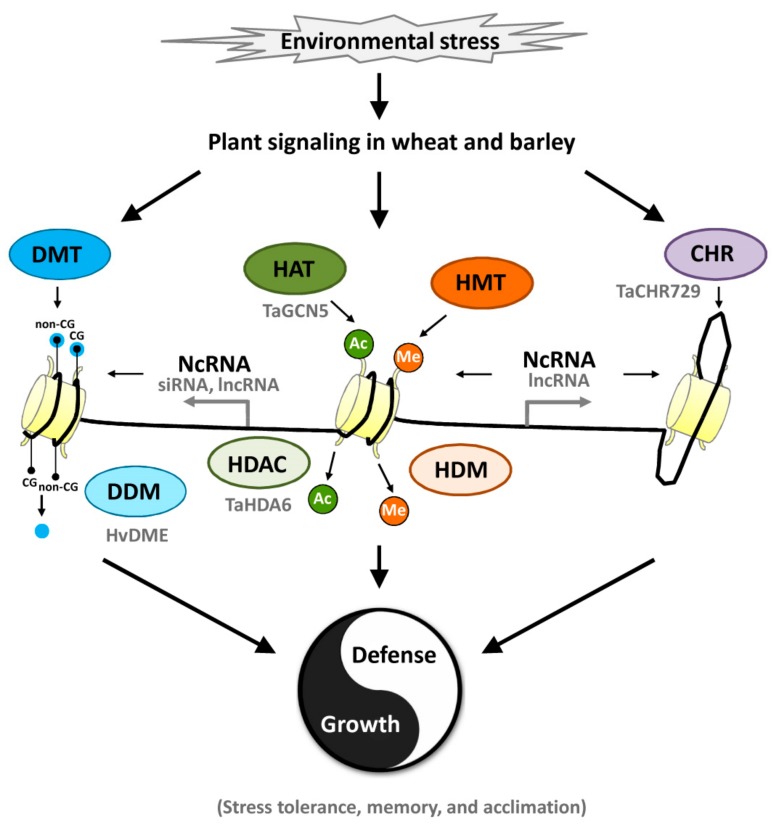
**A general model for the role of epigenetic elements and processes in stress responses in wheat and barley.** DNA methylation is regulated by DMT and DDM, while histone modifications include histone acetylation/deacetyaltion and methylation/demethylation mediated by HAT/HDAC and HMT/HDM enzymes. In addition, CHR-mediated chromatin remodeling and ncRNA-regulated epigenetic processes, including RdDM, histone modification as well as genome topology changes, also regulate gene expression in response to the environmental stresses. Ac, histone acetylation marker; Me, histone methylation marker; CHR, Chromatin remodeling complex/factor; DDM, DNA demethylase; DMT, DNA methyltransferase; HAT, histone acetyltransferase; HDAC, histone deacetylase; HDM, histone demethylase; HMT, histone methyltransferase; IncRNA, long non-coding RNA; NcRNA, non-coding RNA; RdDM, RNA-directed DNA methylation; siRNA, small interfering RNA.

In this review, we discuss the recent advance in the understanding of epigenetic regulation of plant responses to abiotic and biotic stresses in wheat and barley (summarized in Figure 1). Under non-stress condition, the expression of stress responsive genes is repressed. Upon sensing the environmental stresses, plants such as wheat and barley initiate the stress-responsive signaling, which resulted in the epigenetic remodeling involving DNA methylation, histone modification and chromatin remodeling. DNA methylation is regulated by DMT and DDM, while histone modifications include histone acetylation/deacetyaltion and methylation/demethylation mediated by HAT/HDAC and HMT/HDM enzymes. In addition, CHR-mediated chromatin remodeling and ncRNA-regulated epigenetic processes, including RdDM, histone modification as well as genome topology changes, also regulate gene expression in response to the environmental stresses. These epigenetic processes orchestrate the plant stresses responses and fine-tune the balance of plant growth and defense in wheat and barley.

Although past decades have seen progress in understanding the epigenetic mechanisms controlling wheat and barley stress responses, we still have a long way to go towards fully understanding the epigenetic mechanisms regulating plant responses to environmental stresses in wheat and barley. In *Arabidopsis*, the MAP kinase MPK3, a key component in defense signaling, directly phosphorylates the histone deacetylase HD2B, thereby regulating the intra-nuclear compartmentalization of HD2B, as well as the reprogramming of defense gene expression and innate immunity [119]. However, detailed steps from signaling to epigenetic modification in response to environmental stresses remain to be uncovered in wheat and barley. In addition, *Arabidopsis* studies revealed that multiple epigenetic processes such as DNA methylation, histone modifications and chromatin remodeling regulate transcriptional memory to environmental stresses, including heat, freezing, drought and even pathogen infection [120,121,122]. Such stress memory greatly improves plant stress adaptation, and also prepares their offspring for future environmental challenges [120,121,122]. However, stress memory and its epigenetic mechanisms in wheat and barley remain to be explored in the future research.

With the advance of molecular technologies, our knowledge of the mechanisms of epigenetic responses to environmental stresses is rapidly growing, which could certainly lead to the more efficient improvement of cereal crops [123]. In *Arabidopsis*, epigenetic recombinant inbred line (epiRIL) populations were constructed and exhibited discernible phenotypic variation, including altered resistance against pathogen infection [123,124,125,126]. Creating similar epiRILs in wheat and barley would provide substantial resources not only for identifying ideal epigenetic variation in crops, but also for fully using the potential of epigenetics in crop improvement [123,124,125,126]. Besides, genome-editing enzymes such as transcription activator-like effector nucleases (TALENs) and CRISPR-Cas9 system have been used to engineer epigenomes in a sequence-specific manner in mammalian systems [127,128,129,130]. In *Arabidopsis*, Johnson et al. directed DNA methylation to target DNA sequences and caused expected phenotype changes through fusing ZFNs with the SRA domain-containing protein SUVH9—a protein integral to RNA-directed DNA methylation (RdDM) [131]. The development of methodologies to create epiRIL, generate epimutagenesis, and engineer epigenomes in a site-specific manner, would provide new avenues for generating epigenetic diversity and harnessing epigenetic variation for the improvement of agricultural traits in wheat and barley.

## Figures and Tables

**Table 1:** The Epigenetics elements involved in stress response in wheat and barley.

Epigenetic Category	Element Name	Element Category	Species	Biological Function and Evidence	Reference
DNA methylation	HvDME	DNA glysosylase	Barley	*HvDME* expression is induced by drought stress, which is correlated with the differential DNA methylation patterns within the gene.	[55]
Histone modification	TaGCN5	Histone acetyltransferase	Wheat	The expression of the wheat *TaGCN5* gene in Arabidopsis *gcn5* mutant plants complemented the heat and salt tolerance.	[56,57]
	TaHDA6	Histone deacetylase	Wheat	TaHDA6 represses histone acetylation at promoters of defense-related genes and thus negatively regulates their expressions as well as plant defense responses to *Bgt.*	[58]
Chromatin remodeling	TaCHR729	Chromatin remodeling factor	Wheat	TaCHR729 promotes H3K4me3 at the *TaKCS6* promoters and positively regulates the *TaKCS6* expression and cuticular wax biosynthesis, thereby affecting twheat-*Bgt* interaction.	[59]
Non-coding RNA	TalncRNA18, TalncRNA73, TalncRNA106, TalncRNA108	LncRNA	Wheat	They exhibit differential expression and target wheat defense-related genes in response to *Pst* infection	[60]
